# Analytical Pyrolysis of Soluble Bio-Tar from Steam Pretreatment of Bamboo by Using TG–FTIR and Py–GC/MS

**DOI:** 10.3390/ma17091985

**Published:** 2024-04-25

**Authors:** Yongshun Feng, Xin Pan, Hui Qiao, Xiaowei Zhuang

**Affiliations:** Zhejiang Academy of Forestry, Liuhe Road 399, Hangzhou 310023, China; f050914411@163.com (Y.F.); panxin@sohu.com (X.P.);

**Keywords:** moso bamboo, wastewater, soluble bio-tar, TG–FTIR, Py–GCMS

## Abstract

Steam pretreatment at high temperatures enables fresh bamboo to possess antifungal and antiseptic properties. The process produces a large amount of wastewater that urgently needs to be recycled. Soluble bio-tars derived from wastewater under low-temperature (LTS-tar) and high-temperature (HTS-tar) steam pretreatments of moso bamboo were studied with a thermogravimetric analyzer coupled with Fourier transform infrared spectroscopy (TG–FTIR) and pyrolysis–gas-chromatography/mass spectrometry (Py–GC/MS). Thermogravimetric analysis showed that in the three stages of the thermal decomposition process, the final residue of the bamboo and HTS-tar had two main peaks of 0.88 wt% and 6.85 wt%. The LTS-tar had much more complicated thermal decomposition behavior, with six steps and a high residue yield of 23.86 wt%. A large quantity of CH_4_ was observed at the maximum mass loss rates of the bamboo and bio-tars. Acids, aldehydes, ketones, esters, and phenolic compounds were found in the pyrolysis products of the bamboo and soluble bio-tars. Both bio-tars contained carbohydrates and lignin fragments, but the LTS-tar under mild steam conditions had more saccharides and was much more sensitive to temperature. The lignin in the bamboo degraded under harsh steam conditions, resulting in high aromatic and polymeric features for the HTS-tar. The significant differences between LTS-tar and HTS-tar require different techniques to achieve the resource utilization of wastewater in the bamboo industry.

## 1. Introduction

Due to the depletion and environmental issues of traditional fossil resources, various technologies for the utilization of renewable feedstocks have been developed under the framework of biorefinery [1,2,3]. Technologies used for processes such as carbonization, pyrolysis, and gasification produce products that can replace fossil-based fuels, chemicals, and carbon materials. Such thermochemical processes convert biomass into three major products: a solid charcoal-like bio-char, a liquid tar-like bio-oil, and non-condensable gases.

Bio-tar is a byproduct in the conversion process of biomass into solids, liquids, and gases. It is obtained from the thermochemical treatment of lignocellulosic biomass such as harvesting residues and wood processing residues. Bio-tar is a green and earth-friendly alternate to the regular tar that originates from fossil oil. Compared with bio-oil, bio-tar from the gasification of biomass has a lower acid value, water content, and oxygen content but a higher heating value and viscosity. Bio-tar’s special traits make it potentially useful for the extraction of valuable chemicals such as guaiacol, syringol, and cyclotene [4,5]. It is suitable to be used in pitch, varnishes, cements, preservatives, and medicines such as disinfectants and antiseptics [6]. Catalytic cracking can effectively convert bio-tar into green gasoline aromatics [7]. Wood tar from thermal conversion makes it possible to obtain carbon binders with reduced emissions of polycyclic aromatic hydrocarbons [8]. Recently, bio-tar was used to develop porous carbon with high gas uptake capacities for the selective adsorption of CO_2_ from natural gas [9]. A novel N-doped porous carbon from bio-tar was prepared with excellent electrochemical performance [10].

The composition of bio-tar can vary with the kind of biomass and the method of thermochemical conversion [11]. It is a complex mixture derived from the thermal decomposition of biomass that includes single-ring to five-ring aromatic compounds along with polycyclic aromatic hydrocarbons (PAHs). It is composed of various carbon-containing oxygenates that are mainly phenolic compounds but also include acids, alcohols, aldehydes, esters, and ketones [12,13]. The chief constituents are phenol, cresol, catechol, guaiacol, syringol, cyclotene, naphthalene, and other hydrocarbons. In earlier studies, bio-tars were generally considered to be aromatic in nature without a clear distinction between classes of compounds [14]. Many researchers have characterized different kinds of bio-tars. Anca-Couce characterized bio-tar from the torrefaction of spruce. The tar compounds were classified into phenolics, furans, and carbonyls. Pyrolytic lignins with some amounts of sugars were inferred to be components in heavy bio-tars [15]. Song et al. analyzed bio-tar from corn stover, indicating that bio-tar was mainly composed of phenols and polycyclic aromatic hydrocarbons that rapidly decomposed between 183 and 252 °C [16]. Ku et al. separated bio-tars into three fractions by using methanol and ether as extraction solvents; charcoal dust, PAHs, and phenolics with different reactivity were observed in the bio-tars [17]. They further analyzed bio-tars formed during the aging of wood and crude bamboo vinegar with Py–GC/MS, and they found that phenolic compounds were primarily responsible for the formation of bio-tars [18]. An online characterization of bio-tar from beech and pine on a technical scale was developed by using the Laser-Induced Fluorescence (LIF) technique. It was found that two-ring aromatic compounds were the main PAHs in the early stage and three- and four-ring aromatic compounds were the main PAHs in the subsequent stages [19]. A Fourier transform ion cyclotron resonance mass spectrometer (FT–ICR–MS) was adopted to analyze the formation of bio-tars of bio-oil during pyrolysis. Condensed aromatics were formed via the polymerization with one or two rings at low temperatures, while more than four rings were formed at 800 °C [20]. More detailed information on the properties and formation of bio-tar can be found in review articles [21,22,23].

In this study, the analytical pyrolysis characteristics of soluble bio-tars derived from the steam treatment of bamboo were studied. Soluble bio-tars largely exist in the wastewater produced by the steam treatment of bamboo. However, information on such bio-tars from the bamboo industry is very limited, though they are closely related to the stabilization of bamboo wastewater. The characterization of soluble bio-tars derived from the steam pretreatment of bamboo was required to assess the quality of potential bio-tar applications. Understanding the properties and characteristics of bio-tars is crucial for the innocuity treatment and resource utilization of wastewater, which helps reduce production costs and solve environmental issues.

## 2. Materials and Methods

### 2.1. Materials

**Figure 1:**
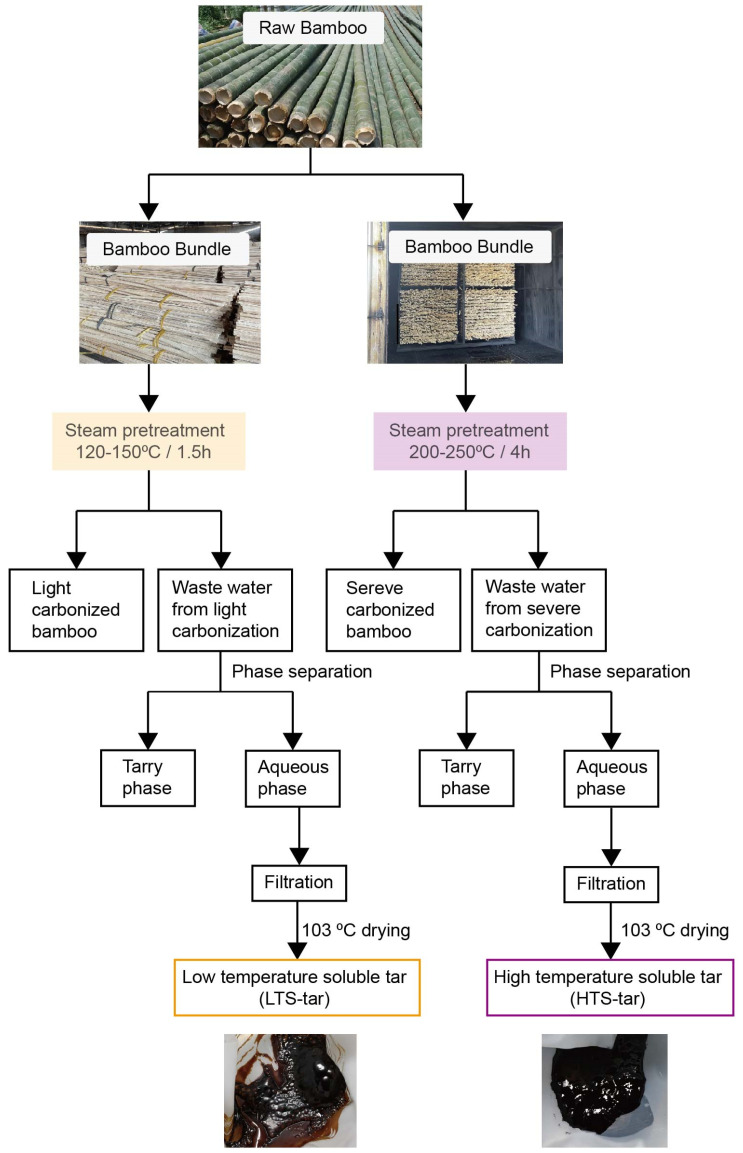
Scheme of the steam pretreatment of moso bamboo.

The materials used for analytical pyrolysis were moso bamboo and soluble tar from the low and high-temperature steam pretreatment of the moso bamboo, as shown in Figure 1. For comparison, the pyrolytic characteristics of the raw moso bamboo were also analyzed. The moso bamboo was directly acquired from the production line of Zhejiang Jiahe Bamboo Technology Company (Lishui, China). The outer and inner layers of the moso bamboo were removed beforehand. It was further ground to 0.1–0.2 mm and dried at 103 °C before analysis. Wastewater from the high-temperature steam pretreatment of the moso bamboo was provided by Zhejiang Jiahe Bamboo Technology Company. This process involved the steam pretreatment of the bamboo at between 200 and 250 °C in a heating chamber at ambient pressure for 4 h. Wastewater from the low-temperature steam pretreatment of the moso bamboo was provided by Zhejiang Chuangwei Bamboo Development Corporation (Quzhou, China). The Chuangwei technology was used in the steam pretreatment of the moso bamboo at between 120 and 150 °C and around 0.2 MPa in a pressurized vessel for 1.5 h. Both types of wastewater were filtered through 200 mesh (74 μm) cloth and dried at 103 °C for 8 h to obtain soluble tar. For nomenclature, soluble tar derived from high-temperature steam pretreatment is referred to as HTS-tar and that derived from low-temperature steam pretreatment is referred to as LTS-tar. The content of the HTS-tar in wastewater accounted for ca. 0.3 wt%, and that of the LTS-tar accounted for ca. 3.0 wt%.

### 2.2. FTIR Analysis of Soluble Bio-Tars in Wastewater

The functional groups of the bamboo and different bio-tars were analyzed with a Fourier Transform Infrared Spectrophotometer (Nicolet iS20, Thermo Fisher Scientific, Waltham, MA, USA). Samples were prepared by tableting the mixture of bamboo or tar with KBr (where a 1–2 mg sample was mixed with 100 mg of KBr and then pressed into a transparent film). The resolution of the FTIR was 4 cm^−1^, and each spectrum was composed of 64 scans. Transmittance was measured with wavenumbers from 650 to 4000 cm^−1^. Before each measurement, the baseline was corrected and the spectra were normalized.

### 2.3. TG–FTIR Analysis of Soluble Bio-Tars in Wastewater

The thermal decomposition behavior of the biomass and its three components were studied using a thermogravimetric analyzer (TA Discovery TGA 55, TA Instruments, New Castle, DE, USA) coupled with Fourier transform infrared spectroscopy (Nicolet iS50, Thermos Fisher Scientific, USA). The Teflon tube and FTIR gas cell were preheated to 180 °C before each experiment. For each test, a 10 mg sample was heated from 30 °C to 850 °C at a linear heating rate of 15 °C /min. The volatile products released during pyrolysis were immediately swept into the FTIR gas cell by the 99.99% pure nitrogen carrier gas at a flow rate of 40 mL/min. The spectrum scope was located in the range of 450–4000 cm^−1^, and the resolution factor was selected to be 1 cm^−1^.

### 2.4. Py–GC/MS Analysis of Soluble Bio-Tars in Wastewater

Py–GC/MS was used to analyze the pyrolysis products of the bamboo and soluble tars. The amount of sample was 0.8 mg for each run, and each sample was pyrolyzed in the pyrolysis apparatus (PY-3030D, Frontier Laboratories Ltd., Fukushima, Japan) at 550 °C by fast pyrolysis. High purity argon was used as the carrier gas with a split ratio of 1:40. The evolved volatiles were analyzed using GC–MS (QP2020 NX, Shimadzu, Kyoto, Japan), where the injector temperature was kept at 250 °C to avoid any condensation of the pyrolysis products. Chromatographic separation was performed with an HP-5MS (30 m × 0.25 mm × 0.25 μm) capillary column. The initial chromatographic column temperature was 50 °C, which was maintained for 5 min; the temperature was then increased at a rate of 10 °C/min until reaching 270 °C. The mass spectra were analyzed in EI mode at 70 eV and obtained from 35 to 450 (*m*/*z*). Chromatographic peaks were identified according to the NIST MS library (NIST 17).

## 3. Results and Discussion

### 3.1. FTIR Spectroscopy Analysis of Soluble Bio-Tars from Low and High-Temperature Pretreatment

**Figure 2:**
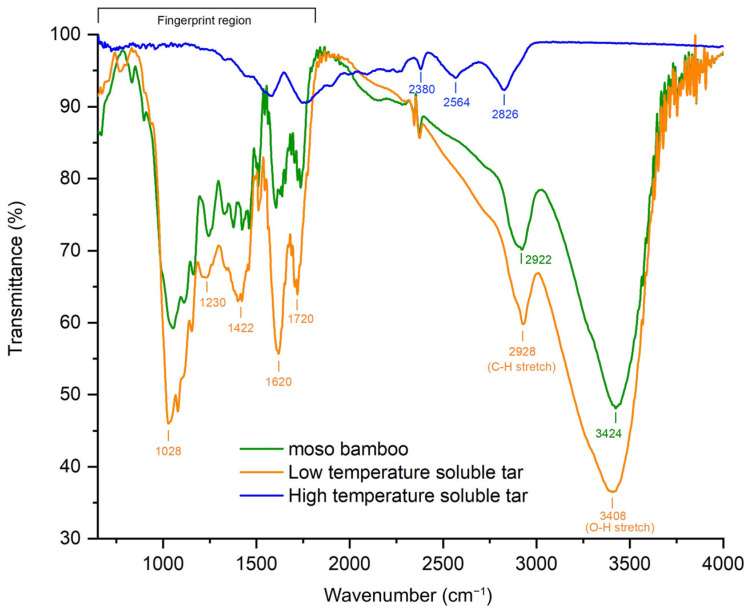
The FTIR spectra of moso bamboo and different soluble bio-tars.

The functional groups of the moso bamboo, LTS-tar, and HTS-tar were characterized based on the FTIR analysis, and their corresponding FTIR spectra are presented in Figure 2. It can be observed that the distribution of major functional groups of the moso bamboo and LTS-tar showed good similarity while the HTS-tar had totally different patterns. For the moso bamboo, the transmission peaks at 3408 cm^−1^ and 2928 cm^−1^ were attributed to O-H and C-H stretching vibrations in carbohydrates, which are commonly found in biomass. The spectra profiles in the fingerprint region (800–1800 cm^−1^) exhibited aromatic properties of the lignin component. C=O stretching in unconjugated ketone, carbonyl, and ester groups were found in the range of 1709–1738 cm^−1^ peaks. Peaks at 1505–1605 cm^−1^ showed aromatic skeleton vibrations, and peaks at 1376 cm^−1^ were attributed to the aliphatic C-H stretching in -CH_3_ and phenols. Peaks in the range of 1214–1365 cm^−1^ showed the C-C, C-O, and C=O stretching of guaiacol and syringol rings, and peaks in the range of 1030–1166 cm^−1^ showed aromatic C-H deformation in guaiacol and syringol units.

It was found that the LTS-tar had a similar FTIR pattern to the moso bamboo. However, the peak intensities of the LTS-tar were much higher than those of the moso bamboo, especially in the fingerprint region, showing stronger aromatic features. The LTS-tar originated from the steam pretreatment of the moso bamboo at 120–150 °C for 1.5 h. No significant degradation of cellulose, hemicellulose, and lignin in the moso bamboo occurred at such a low temperature, but small amounts dissolved in the hot steam, leading to the similarity of the functional groups for the LTS-tar and moso bamboo. There were no obvious transmittance peaks of aromatic groups for the HTS-tar, as the steam conditions for the HTS-tar were much harsher at 200–250 °C for 4 h. The functional groups of the main organic compounds disappeared in the HTS, as they decomposed during the high-temperature steam treatment process. The main components in the moso bamboo were degraded, leaving more fixed carbon in the soluble bio-tar.

### 3.2. TG/DTG Analysis of Soluble Bio-Tars from Low and High-Temperature Pretreatment

**Figure 3:**
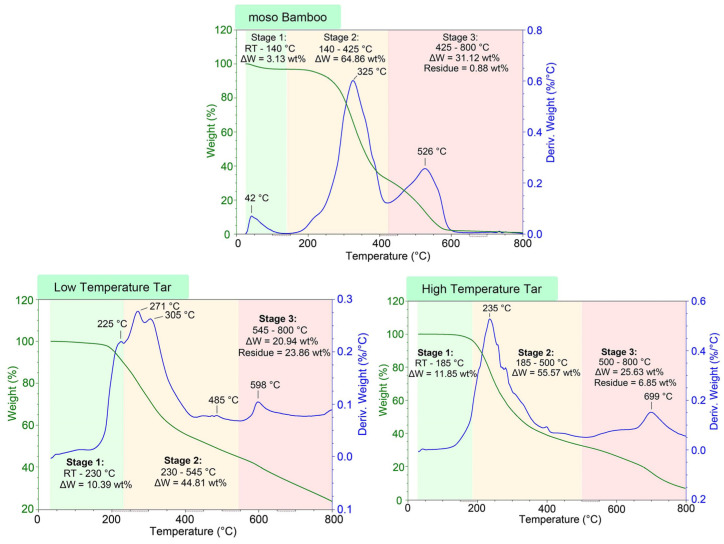
DT/DTG curves of moso bamboo, low-temperature soluble tar, and high-temperature soluble tar.

The thermal behavior (TG and DTG curves) of the moso bamboo and two different soluble bio-tars during pyrolysis is shown in Figure 3. The moso bamboo and its soluble bio-tars showed totally different pyrolysis properties. In general, the pyrolysis process of the moso bamboo and two different soluble bio-tars was divided into three stages according to the weight loss rate. For comparison, the thermo-degradation property of the moso bamboo was first analyzed. As a kind of typical biomass, the three pyrolysis stages of moso bamboo are similar to other biomasses. The percent of mass decreased by about 3 wt% below 140 °C due to the release of bound water in the sample [24]. The thermal decomposition of organic composition was therefore deemed to have started at 140 °C and including two main peaks, shown in Figure 3. In the temperature range of 140 °C and 425 °C, the degradation of hemicellulose first took place, followed by cellulose and lignin with a weight loss of 64.86 wt%. Jiang et al. also confirmed a 68.69 wt% mass loss of moso bamboo pyrolysis at between 177 °C and 377 °C [25]. Wang et al. summarized the mechanisms of cellulose, hemicellulose, and lignin pyrolysis, indicating that the peak temperature of hardwood and straw is in the range of 274–291 °C. The minimum temperature for the decomposition of cellulose crystals is greater than 300 °C, and the glass transition temperature of amorphous cellulose is 243–307 °C [26]. Our DTG results showed that the peak temperature of the moso bamboo was 325 °C, resulting from the overlapping decomposition of hemicellulose and cellulose, with the latter playing a more important role. Lignin decomposition occurred over a wider temperature range. The β-O-4 linkages in lignin can be cleaved at 200–250 °C, and the decomposition of other linkages and side chains lasts until 800 °C. The degradation of the stable lignin of the moso bamboo in the third stage emerged with a weight loss peak at 526 °C. The decomposition of the moso bamboo was generally completed before 650 °C. Weight loss in the temperature range of 650–800 °C could be neglected. The residue that remained in the crucible was as low as 0.88 wt% of the original moso bamboo [25], as the inner and outer skin of the moso bamboo used in this study had already been removed.

The pyrolysis characteristics of the LTS-tar were much more complicated than those of the moso bamboo and HTS-tar, although both bio-tars originated from the steam pretreatment of the moso bamboo. In the three stages of the LTS-tar pyrolysis process, there were six steps with temperature boundaries of 230, 290, 430, 545, and 700 °C, as shown in Figure 3. According to the DTG curve of the LTS-tar, the first peak appeared at 225 °C and the highest peak occurred at 271 °C, followed by another peak at 305 °C. Most of the mass loss occurred in the temperature range of room temperature to 430 °C, with 46.56 wt% of the weight loss. The weight loss rate in 430–800 °C was not so fierce, with a mass loss of 29.58 wt%. The final residue accounted for 23.86 wt% of the total mass. The relatively increased number of pyrolysis steps but narrow temperature range indicated that the chemical composition of the LTS-tar was much more complicated. The steam pretreatment of the moso bamboo at mild conditions (120–150 °C for 1.5 h) led to the decomposition of hemicellulose while the structure of cellulose remained unchanged. Thus, the steam pretreatment resulted in the release of various saccharides from the degradation of hemicellulose, such as mannose, glucose, galactose, arabinose, and xylose. These monosaccharides were water-soluble and underwent no further cracking at mild conditions. The LTS-tar had a large amount of these saccharides and therefore reached a relatively high level of 3.0 wt% in the wastewater.

Meanwhile, the three pyrolysis stages of the HTS-tar did not occur several small steps. Stages 1 and 2 were from room temperature to 500 °C, with the maximum weight loss rate at 235 °C, which was much lower than that of the moso bamboo and LTS-tar. Water no longer existed in the HTS-tar. The HTS-tar was condensed from the harsh steam pretreatment (200–250 °C, 4 h) of the moso bamboo. The color of the moso bamboo changed from light yellow to dark brown after this process. During this process, monosaccharides and even amorphous cellulose encountered ring-open reactions and degraded into smaller molecules such as acids, ketones, and furans. They reached the relatively low amount of 0.3 wt% in the wastewater, and the mass loss reached 67.52 wt% below 500 °C. The second peak in the DTG curve appeared at 699 °C, which was probably due to the cracking of lignin-based polymers and the degradation of fixed carbons.

### 3.3. Evolution of Released Gases from Pyrolysis of Soluble Bio-Tars from Low and High-Temperature Pretreatment

**Figure 4:**
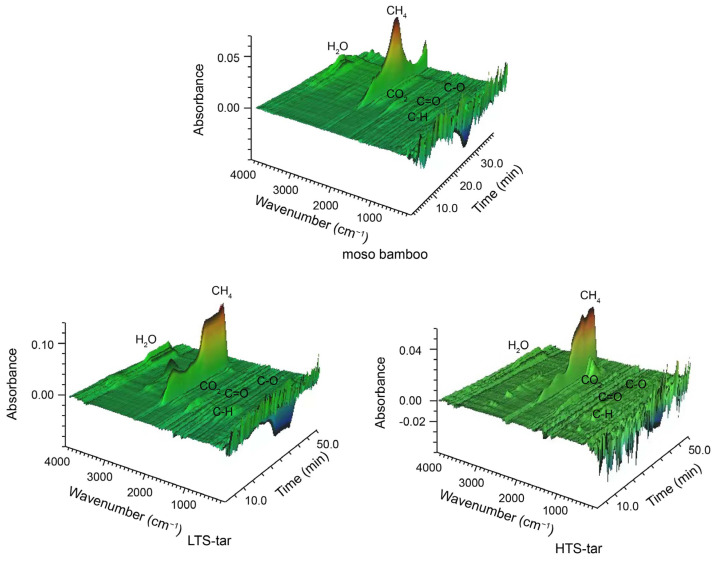
3D infrared spectrum of the evolution gases of bamboo and soluble bio-tars.

The infrared spectrum is often used to distinguish various inorganic and organic compounds during the pyrolysis process. Commonly detected pyrolysis products included non-condensable gases, such as CO, CO_2_, and CH_4_, and condensable volatiles, such as H_2_O, methanol, acids, and phenols [25]. Figure 4 shows a three-dimensional plot of the spectra obtained from the evolved gases during the pyrolysis of the moso bamboo and two different soluble bio-tars. The height of absorbance qualitatively reflects the concentration of the product in the volatile mixture. The main gaseous products and chemical linkages identified in the spectra included H_2_O, CH_4_, CO_2_, C-C, C=O, and C-O. The C=O linkage mainly existed in the carboxylic acid and esters, while the C-O linkage appeared in the alcohols. The compositions of the mixed volatile gases from the moso bamboo and bio-tars were different from each other, with different evolving characteristics.

The most abundant gas for all samples was CH_4_, which was found to correspond to the mass loss rate peaks obtained from the thermogravimetry analysis of this study. The fixed carbon content in the tar is higher than that of the moso bamboo. This resulted in a longer CH_4_ release, which was reflected as a peak platform in the bio-tars. The previous FTIR results showed that both the moso bamboo and LTS-tar contain carbohydrates and lignin, although at different proportions. Therefore, the stretching vibrations of specific functional groups showed a good similarity between the moso bamboo and LTS-tar. The main released gases in the HTS-tar were CH_4_ and CO_2_. The peaks of the HTS-tar in the fingerprint region of the FTIR spectra were more complicated, indicating that the aromatic units in lignin underwent rearrangement during the high-temperature steam pretreatment.

### 3.4. Py–GC/MS Analysis of Soluble Bio-Tars from Low and High-Temperature Pretreatment

**Figure 5:**
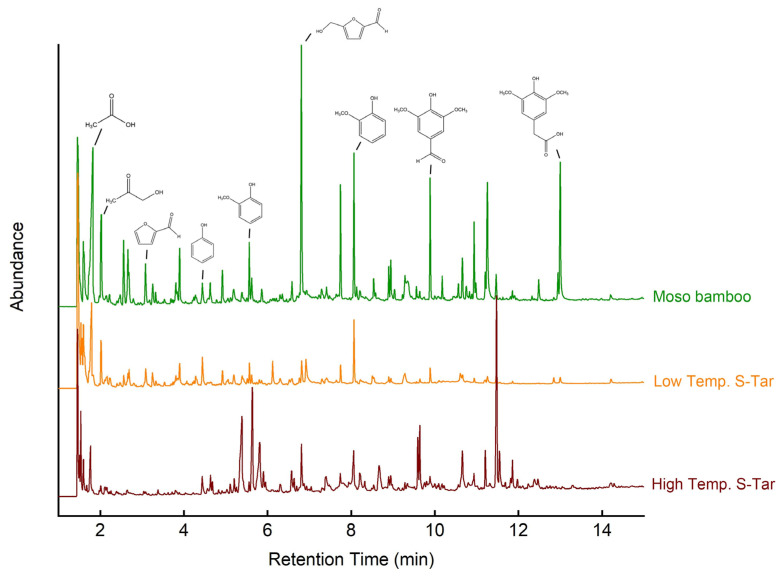
Total ion chromatograms of moso bamboo and soluble bio-tars.

Py–GC/MS is a convenient tool to quantitatively investigate the product distribution of bio-based materials. The pyrolytic vapors generated from the solid samples in the micro-pyrolyzer were directly transported into the GC/MS for compositional characterization without condensation. The total ion chromatograms (TICs) of the moso bamboo and soluble bio-tars are shown in Figure 5. The total ion chromatographs of the three samples are quite different from each other, indicating that they had different compositions. The pyrolysis products of the moso bamboo were found to contain acids, ketones, furans, and aromatics as the main thermal degradation products, which is in accordance with the pyrolysis products of biomass composed of cellulose, hemicellulose, and lignin [25]. Detailed composition and retention time information is presented in Table 1.

**Figure 6:**
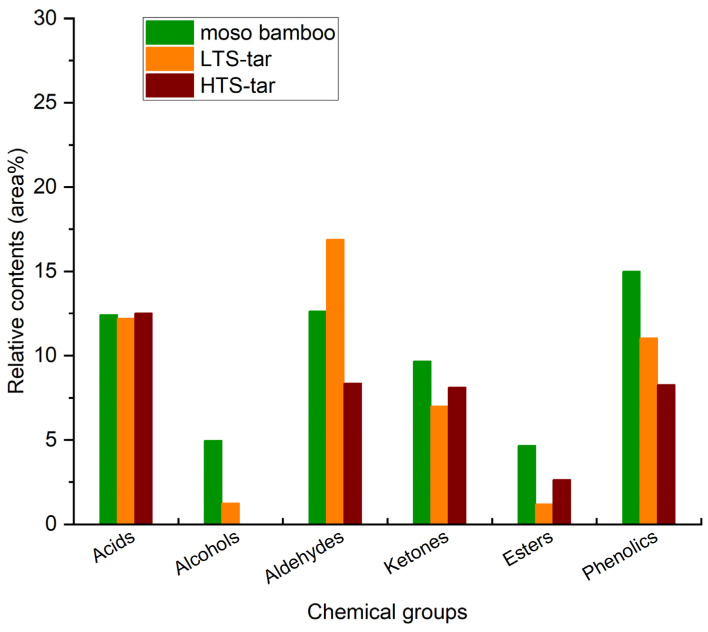
Distribution of pyrolysis compounds of moso bamboo and soluble bio-tars.

The organic chemicals analyzed in the pyrolysis products from the moso bamboo and bio-tars were divided into eight groups: acids, alcohols, aldehydes, ketones, esters, phenolics, sugars, and others. The relative contents of the chemical groups are shown in Figure 6. The most abundant single compound was acetic acid, which accounted for 11.59%, 11.34%, and 2.26% of the moso bamboo, LTS-tar, and HTS-tar. Acetic acid is commonly identified as a main component, with concentrations up to 10% in bio-oil, and it is a good building block for chemical production [4]. However, the HTS-tar in this study contained 10.27% 4-hydroxy-3,5-dimethoxy benzene acetic acid, whereas the other two samples contained less than 1%. This was probably due to the chemical reaction of the phenolic compounds and acids under high temperatures during extended steam pretreatment. Alcohols were found in both the moso bamboo and LTS-tar, as they had both carbohydrates, which was also confirmed by previous FTIR results.

Aldehydes and ketones with C=O linkages are the main chemical groups in pyrolysis biomass products. They were also found as main compounds in the pyrolysis products of this study’s moso bamboo and bio-tars. The contents of aldehyde were 12.63%, 16.89%, and 8.36% and the contents of ketones were 9.66%, 7.01%, and 8.12% for the moso bamboo, LTS-tar, and HTS-tar, respectively. The aldehydes mainly included butanedial, hydroxybenzaldehyde, furfural, and derivatives from furfural. Ketones have various types of chemical structures and formulas, such as hydroxyacetone, ethenone, and cyclopentenone. Esters were also found in the pyrolysis products, though in relatively low amounts, namely, contents of less than 5%.

Phenolic compounds were found in all samples, with the highest amount in the moso bamboo at a content of 15.00%, followed by the LTS-tar at a content of 11.04% and the HTS-tar at a content of 8.28%; see Figure 6. Phenolic compounds include phenol, guaiacol, syringol, and their derivatives that are degraded from lignin in moso bamboo or aromatic polymers in bio-tar [21]. Table 1 shows that the HTS-tar had a much larger amount of GC-unknown compounds in comparison to the moso bamboo, indicating that the composition of the HTS-tar contained high levels of aromatic and polymeric features.

In summary, the pyrolysis properties and chemical compositions of soluble bio-tars showed totally different characteristics depending on the pretreatment time and temperature. The bio-tar derived from the low-temperature steam pretreatment had more organic compounds, which makes it suitable for the extraction of valuable chemicals. On the contrary, the bio-tar derived from the high-temperature steam pretreatment is more suitable for the development of carbon-based materials.

## 4. Conclusions

Temperature plays an important role in the steam pretreatment of raw moso bamboo under 300 °C. It not only influences the quality of the treated bamboo but also leads to totally different wastewater properties. In this work, the pyrolytic characteristics of soluble tar in two different types of bamboo wastewater along with raw moso bamboo were investigated with TG–FTIR and Py–GCMS. The process of thermal decomposition was divided into three stages for the bio-tars and bamboo, six steps for the low-temperature soluble tar, and two steps for the high-temperature tar. At the temperature of the maximum weight loss rate, the gases released by the bamboo and tar were mainly CH_4_ and CO_2_. The low-temperature soluble tar contained more thermal-sensitive components. Acids, aldehydes, ketones, and phenolic compounds were found in the pyrolysis products of the bamboo and different bio-tars. The soluble tar from the low-temperature steam pretreatment contained a certain amount of the same carbohydrate components as the raw bamboo. The aromatic units in the lignins did not change much under low-temperature (120–150 °C) steam pretreatment but underwent rearrangement and polymerization under high-temperature (200–250 °C) conditions. The bio-tars could be modified into high-value carbons with applications in energy storage materials. Regarding the significant differences between soluble tar types in wastewater from the bamboo industry, different resource utilization strategies should be developed.

## Figures and Tables

**Table 1:** Pyrolysis products of moso bamboo and soluble bio-tars according to Py–GC/MS.

R.T. (min)	Name of Compound	Formula	Bamboo (Area %)	LTS-Tar * (Area %)	HTS-Tar * (Area %)
1.456	Carbon dioxide	CO_2_	10.09	25.60	6.56
1.502	Acetaldehyde	C_2_H_4_O	/	/	1.57
1.538	Trimethyl amine	C_3_H_9_N	/	6.77	2.89
1.594	Isopropenyl acetate	C_5_H_8_O_2_	1.41	/	/
1.597	Butanedial	C_4_H_6_O_2_	2.41	9.03	1.59
1.789	Acetic acid	C_2_H_4_O_2_	11.59	11.34	2.26
2.023	Hydroxyacetone	C_3_H_6_O_2_	3.97	3.81	0.45
2.234	2,5-Dimethylfuran	C_6_H_8_O	/	1.38	/
2.559	1,2-Ethanediol,1-acetate	C_4_H_8_O_3_	2.26	/	/
2.687	Methyl pyruvate	C_4_H_6_O_3_	0.99	1.19	/
3.082	Furfural	C_5_H_4_O_2_	1.57	2.17	/
3.249	Furfuryl alcohol	C_5_H_6_O_2_	/	1.25	/
3.809	Dihydropyran	C_5_H_8_O	/	1.05	/
4.284	5-Methyl furfural	C_6_H_6_O_2_	/	1.15	/
4.442	Phenol	C_6_H_6_O	0.92	2.59	1.11
4.638	Pyridine, 3-methoxy-	C_6_H_7_NO	/	/	1.00
4.921	Methyl cyclopentenolone	C_6_H_8_O_2_	1.04	/	/
4.923	3-Methyl-1,2-cyclopentanedione	C_6_H_8_O_2_	/	1.06	/
5.196	N-Carbobenzyloxy-L-glutamine	C_13_H_16_N_2_O_5_	/	1.06	/
5.387	Isobutyric anhydride	C_8_H_14_O_3_	/	/	7.60
5.388	p-Cresol	C_7_H_8_O	0.49	1.59	/
5.564	Guaiacol	C_7_H_8_O_2_	1.58	1.40	0.44
5.623	Cyclopropyl carbinol	C_4_H_8_O	0.85	0.80	6.24
5.812	3-Hydroxypyridine	C_5_H_5_NO	/	/	5.71
6.123	4H-Pyran-4-one,2,3-dihydro-3,5-dihydroxy-6-methyl-	C_6_H_8_O_4_	/	2.14	/
6.811	2,3-Dihydrobenzofuran	C_8_H_8_O	8.79	2.05	2.82
6.919	5-Hydroxymethylfurfural	C_6_H_6_O_3_	/	3.45	/
7.399	Hydroquinone	C_6_H_6_O_2_	/	/	1.64
7.741	Ethanone,1-(4-hydroxy-2-methylphenyl)-	C_9_H_10_O_2_	/	/	1.66
7.746	4-Hydroxy-3-methoxystyrene	C_9_H_10_O_2_	3.19	1.25	/
8.068	2,6-Dimethoxyphenol	C_8_H_10_O_3_	4.23	4.20	3.50
8.208	3-Hydroxybenzaldehyde	C_7_H_6_O_2_	/	/	1.80
8.67	Amyl acetate	C_7_H_14_O_2_	/	/	2.65
8.948	iso-Eugenol	C_10_H_12_O_2_	1.30	/	/
9.288	Benzenethiol,4-(1,1-dimethylethyl)-	C_10_H_14_S	1.23	/	/
9.347	1,6-Anhydro-β-d-glucopyranose	C_6_H_10_O_5_	1.68	/	/
9.641	2-Propanone,1-(4-hydroxy-3-methoxyphenyl)-	C_10_H_12_O_3_	0.28	/	2.79
9.89	3′,5′-Dimethoxyacetophenone	C_10_H_12_O_3_	3.37	/	/
10.661	Syringaldehyde	C_9_H_10_O_4_	1.33	/	3.40
10.938	Methoxyeugenol	C_11_H_14_O_3_	2.08	/	1.59
11.21	Acetosyringone	C_10_H_12_O_4_	1.00	/	3.22
11.258	Coniferol	C_10_H_12_O_3_	4.97	/	/
11.469	Benzeneacetic acid,4-hydroxy-3,5-dimethoxy-	C_10_H_12_O_5_	0.84	0.87	10.27
13.002	5,6-Dimethoxyphthalaldehydic acid	C_10_H_10_O_5_	5.29	/	/
Unknown			21.28	12.81	27.26

*: LTS-tar: low-temperature soluble tar; HTS-tar: high-temperature soluble tar. “/”: trace amount or not detected by Py–GC/MS.

## Data Availability

Data are contained within the article.
